# Mathematical Modeling and Release Kinetics of Green Tea Polyphenols Released from Casein Nanoparticles

**DOI:** 10.22037/ijpr.2019.1100715

**Published:** 2019

**Authors:** Ravi Theaj Prakash Upputuri, Abul Kalam Azad Mandal

**Affiliations:** *School of Bio Sciences and Technology, Vellore Institute of Technology, Vellore-632014, Tamil Nadu, India.*

**Keywords:** Anomalous transport, Casein nanoparticles, Green tea polyphenols, Mathematical modeling, Sustained release, Zero order kinetics

## Abstract

Drug release kinetics plays an important role in determining the mechanism of drug release, which in turn helps in formulating controlled/sustained release formulations. In our study, different concentrations of green tea polyphenols (GTP) were encapsulated into casein nanoparticles which showed a maximum encapsulation efficiency (76.9%) at a GTP concentration of 5 mg/mL. The casein nanoparticles were characterized through particle size analysis, zeta potential, AFM, and HR SEM, followed by molecular docking studies, which confirmed the binding of GTP to casein nanoparticles. *In-vitro* release studies carried out at different temperatures and pH showed no significant difference in the release pattern, but the release was prolonged even up to 48 h. On varying pH of the release medium, an increase in the percentage of release was observed as the pH shifted from acidic to basic. All release data showed good correlation with Zero order kinetics, an ideal model for release of drugs from nanoparticulate sustained release formulations, with anomalous mode of drug transport. Antioxidant activity of the released GTP determined through DPPH assay showed potent antioxidant effect of GTP even after 48 h of its release. Our data indicated that casein nanoparticles could be used as a potent vehicle for the delivery of GTP for achieving a sustained release.

## Introduction

Among many applications of biodegradable polymers, one of the important applications is in the area of sustained and controlled drug delivery, due to low cytotoxicity and higher tissue compatibility (1, 2). Proteins, being a versatile class of biopolymers, are widely studied for their application in nanoparticulate drug delivery systems. Casein, a major milk protein, due to a number of interesting properties, is regarded as a good candidate for drug delivery system. The efficiency of casein as a nanocarrier for drug delivery has been reviewed by Elzoghby *et al*. (3). In the recent past, casein based nano delivery systems are being developed for the delivery of drug and nutraceuticals varying from nano sized micelles to nanoparticles prepared by polyelectrolyte, copolymerization, or ionic complexion (3). Also, casein has a tendency to associate with other bioactives, which is applicable to the process of nanoencapsulation (4). Green tea polyphenols (GTP) are known for its medicinal properties. Various forms of catechins such as epicatechin (EC), epigallocaetchin (EGC), epicatechin-3-gallate (ECG), and epigallocatechin-3-gallate (EGCG) constitute major part of polyphenols. Among all these forms, epigallocatechin-3-gallate (EGCG) constitutes for 50-80% of total catechins (5). There are several reports on the applications of GTP in the prevention of cancer, cardiovascular diseases, neurodegeneration, and diabetes (6-10). GTP is also a strong antioxidant, and has psychotropic effect (11, 12). In spite of these health benefits, GTP has several limitations with regard to bioavailability, stability, and biotransformation (13-15). To overcome these limitations, nanoparticulate formulations have been prepared to maximize the health benefits of GTP. 

The time required for the new delivery device could be reduced if the mechanism of drug release is known (16). Mathematical modelling of the *in-vitro* release data plays an important role in providing tools to analyze the experimental data in which the formulation and design factors influence the release data (17). To the best of our knowledge, no reports have been published on encapsulation of GTP into casein nanoparticles. In this study casein nanoparticles have been used to encapsulate GTP. *In-vitro* studies have been conducted to achieve a sustained release and the mechanism of drug release was determined through mathematical modelling.

## Experimental

Green tea polyphenols (GTP) and glutaraldehyde were purchased from Sigma Chemical Co., USA. Casein and other chemicals were obtained from HiMedia, India.

*Preparation of casein nanoparticles*

Casein nanoparticles were prepared by cross linking the particles with glutaraldehyde using desolvation step. About 200 mg of casein was dissolved in 2 mL of 1 N NaOH. The solution was stirred continuously and 8 mL of ethanol was added dropwise at a constant rate of 1 mL/min which resulted in the formation of casein nanoparticles. The particles formed were stabilized by the addition of 8% glutaraldehyde (1.175 μL/mg casein). The cross linking was performed for about 24 h at room temperature under constant stirring through a magnetic stirrer. For the preparation of GTP loaded casein nanoparticles, 1 to 10 mg/mL GTP was added to the reaction mixture prior to cross linking, followed by the addition of glutaraldehyde. The mixture was centrifuged at 8000 rpm for 20 min at 4 ºC. The pellet contained nanoparticles while the supernatant was used for quantification of unloaded GTP. 

*Characterization of casein nanoparticles*

Mean diameter and surface charge of the nanoparticles were determined through dynamic light scattering and ζ potential analysis. Analysis was carried out using Nanopartica, Nanoparticle analyzer SZ-100. Morphology and polydispersity of the particles were analyzed through AFM (Nano Surf Easy Scan2, Switzerland) and HR SEM (FEI Quanta FEG 200 – High Resolution Scanning Electron Microscope). 

Major constituents of tea polyphenols (EGCG, ECG, EGC, EC and (+) Catechin) were docked with the target protein, casein to confirm its potential of binding. The docking analysis was carried out using Auto dock tools (ADT) v1.5.4 and AutoDock v4.2 programs (18). The search was extended over the whole receptor protein used as blind docking.

*Determination of encapsulation efficiency* *(EE)*

Estimation of GTP in the supernatant was carried out by Folin-Ciocalteu assay as described by Swain and Hillis (19). Briefly, 0.5 mL of sample was mixed with 0.5 mL Follin - Ciocalteu reagent (1 M) and 0.5 mL sodium carbonate (35%) and incubated in dark for 30 min. Absorbance was recorded at 700 nm in a spectrophotometer. The concentration of GTP was calculated from standard curve of GTP. All experiments were performed in triplicates. Encapsulation efficiency (EE) was calculated using the formula:

EE (%) = {(Amount of GTP - Amount of free GTP)/Total amount of GTP} × 100

*Drug release profile of GTP loaded casein nanoparticles*

About 1 mL of GTP loaded nanoparticles were placed in a tube containing 10 mL phosphate buffer saline (PBS), pH 7.4 at 37 °C. At regular intervals (1, 2, 3, 4, 5, 6, 7, 8, 12, 24 and 48 h), one mL of sample from the tube was withdrawn and equal volume of fresh PBS was added. The samples were centrifuged and the supernatant was used to determine the amount of GTP released. The estimation of GTP was carried out by the method of Swain and Hillis (19). The drug release studies were carried out for different concentrations of GTP (1, 2.5, 5 and 10 mg/mL), at different pH conditions (pH 3, 7.4 and 9) and at different temperatures (25, 37 and 45 ºC). The drug release kinetics and the mechanism of release were determined for each profile.

*Mathematical modelling and release kinetics*

To study the mechanism of drug release, the *in-vitro* release data was fitted in various mathematical models like Zero order, First order, Higuchi, Hixson–Crowell and Korsemeyer- Peppas models (20). The correlation coefficient (R^2^) was used as an indicator for the best fit for each of the models and release exponent (n) to determine the mechanism of release (Table 1).

*Antioxidant assay*

Radical scavenging activity (antioxidant activity) of released GTP was determined *in-vitro* by 1, 1-diphenyl-2-picrylhydrazyl (DPPH) assay following the method reported by Williams *et al*. (21). DPPH was prepared in absolute ethanol at a concentration of 0.1 mM. About 100 μL sample was added to 550 μL PBS (pH 7.4) and 0.5 mL of DPPH. The reaction mixture was incubated in dark at room temperature for about 30 min and the absorbance was recorded at 517 nm in UV-visible spectrophotometer against control solution. The experiment was repeated three times.

*Statistical analysis*

All data analysed are presented as mean ± SD. The means were separated by Duncan’s multiple range test (DMRT) at *p ≤ *0.05.

## Results

**Figure 1:**
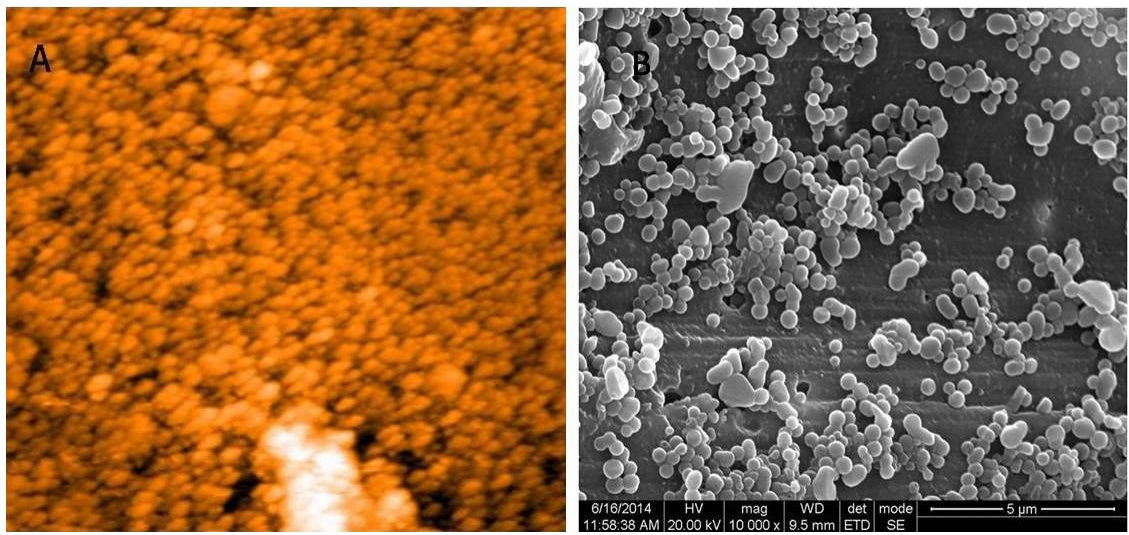
(A) AFM and (B) HR SEM images of casein nanoparticles. Morphology of the particles was observed to be spherical with a diameter of 63 and 97 nm respectively

Casein nanoparticles were prepared through desolvation technique. The casein nanoparticles were incubated in aqueous solution of GTP ranging from 1 to 10 mg/mL to determine the optimum loading concentration of GTP. AFM and HR SEM images showed spherical nanoparticles with sizes of 63 and 97 nm respectively (Figure 1). Average size of the synthesised casein nanoparticles was observed to be 275.4 nm through particle size analyser.

Surface charge of the particles was determined for both unloaded and GTP loaded casein nanoparticles. Unloaded nanoparticles were negatively charged with an electric potential of -11.7 mV. Loading of GTP resulted significant increase in the charge of the particles and was observed to be -29.7, -24.5, -24.2 and -22.9 mV when GTP was loaded at a concentration of 1, 2.5, 5 and 10 mg/mL respectively (Table 2).

**Figure 2:**
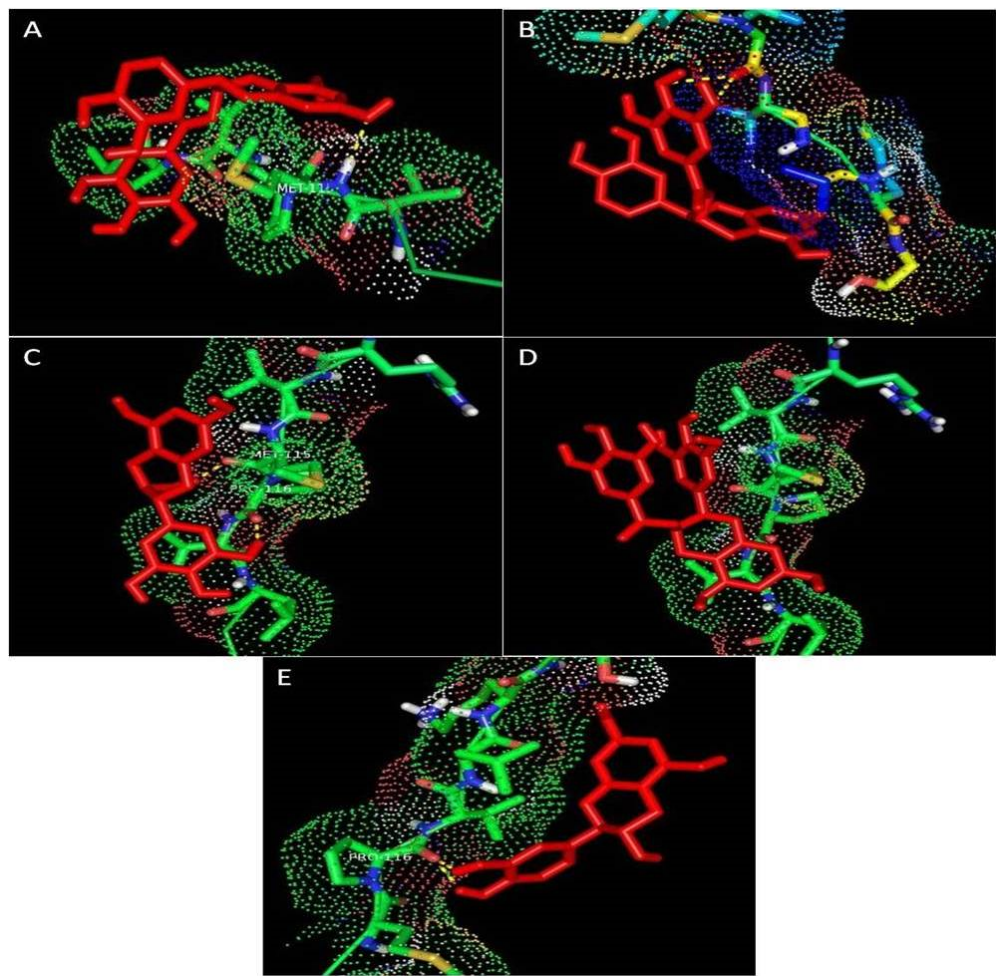
Docked orientation of ligands with the target protein. Interaction between casein and (A) EC, (B) ECG, (C) EGC. (D) EGCG, (E) (+) - catechin

The interactions between the protein and the ligands were determined through molecular docking studies. The docked orientation of ligands with the target protein is shown in Figure 2 and the hydrophobic interactions and the docked amino acids are presented in Table 3. Highest interactions were observed with casein and ECG and the least interactions between casein and EGCG with no hydrogen bonds formed and a much weaker bond was observed with a bonding energy of -1.67 kcal/mol.

**Table 1:** Equations for various kinetic models and their parameters

**Model**	**Equation**	**Parameters definition**
Zero order	C = kot	C: Concentration of drug ko: Zero order constant t: Time
First order	logCo - logCt = k1t/2.303	Co: Initial concentration Ct: Concentration at time t k1: First order constant t: Time
Higuchi Model	Q = k t1/2 H	Q: Amount of drug released in time t: Time kH: Higuchi constant t: Time
Hixson Crowell Model	Q 1/3 - Q 1/3 = k t o t HC	Qo: Initial amount of drug Qt: Remaining amount of rug at time t kHC: Hixson Crowell constant t: Time
Korsmeyer-Peppas Model	M - M = ktn t α	Mt - Mα: Fraction of drug released at time t k: Korsmeyer-Peppas constant t: Time n: Release exponent

**Table 2:** Hydrodynamic diameter, polydispersity index (PDI), ζ potential and encapsulation efficiency (%) (EE), of GTP loaded casein nanoparticles

**Conc. of GTP (mg/mL)**	**Hydrodynamic diameter (nm)**	**PDI**	**ζ potential (mV)**	**EE (%)**
1.0	77.3 ± 15.65d	0.532 ± 0.15b	-29.7 ± 1.23a	65.409 ± 2.151c
2.5	313.4 ± 9.87b	0.178 ± 0.17d	-24.5 ± 0.27b	71.101 ± 0.133b
5.0	275.4 ± 9.15c	0.276 ± 0.20c	-24.2 ± 0.52b	76.945 ± 2.935a
10.0	428.1 ± 13.79a	2.591 ± 0.35a	-22.9 ± 1.02b	70.261 ± 0.953b

Data represented as mean ± SD (n = 3). Different letters (a, b, c, and d) indicate significant difference within the group according to DMRT, *p *≤ 0.05.

**Table 3:** Hydrophobic interactions and docked amino acid residues of albumin with EC, ECG, EGC, EGCG and (+)- catechin

**Ligand**	**Protein**	**Binding Energy**	**Ligand efficiency**	**Intermole energy**	**Ligand atoms**	**Docked amino** **Acid residue(bond length)**
EC	Casein	-3.25	-0.1	-4.79	C-7’ OH	A-Met 115/HN (2.9 A⁰)
ECG	Casein	-3.44	-0.11	-5.15	D-6’ OH D-7’ OH	A-Pro 116/O (2.6, 2.2 A⁰)
EGC	Casein	-3.39	-0.15	-4.25	A-3’OH B-4’ OH	A-Met 115/O (1.8 A⁰) A-Pro 116/O (1.7 A⁰)
EGCG	Casein	-1.67	-0.05	-3.88	-	A-Ser 120/HN
**(+)-**Catechin	Casein	-3.3	-0.16	-4.38	B-2’ OH B-3’ OH	A-Pro 116/O (2.0, 2.1 A⁰)

**Table 4:** GTP release profiles with various models at different loading concentrations of GTP and at different conditions

**Condition**	**Zero order**		**First order **		Higuchi model		Hixson- Crowell model		**Korsmeyer- Peppas model**		**Drug transport mechanism**
	**R²**	**k₀**	**R²**	**k** **1**	**R²**	**k** **H**	**R²**	**k** **HC**	**R²**	**n**	
1 mg/mL GTP	0.9901	0.501	0.7792	0.019	0.8106	5.49	0.898	0.002	0.859	0.641	Anomalous
2.5 mg/mL GTP	0.9807	0.892	0.9879	0.023	0.7529	9.78	0.9922	0.002	0.8395	0.438	Fickian
5 mg/mL GTP	0.9964	1.321	0.9829	0.015	0.7699	17.7	0.9936	0.053	0.8548	0.548	Anomalous
10 mg/mL GTP	0.9970	1.717	0.9577	0.011	0.7868	26.1	0.9842	0.001	0.8613	0.573	Anomalous
pH 3	0.9794	0.730	0.9537	0.018	0.8432	9.80	0.9649	0.059	0.8483	0.674	Anomalous
pH 7.4	0.9964	1.321	0.9829	0.015	0.7699	17.7	0.9936	0.053	0.8548	0.548	Anomalous
pH 9	0.9956	1.52	0.9534	0.014	0.7912	20.3	0.9779	0.051	0.8616	0.636	Anomalous
RT	0.9912	1.21	0.9407	0.010	0.8385	18.8	0.9634	0.038	0.8844	0.647	Anomalous
37 °C	0.9964	1.321	0.9829	0.015	0.7699	17.7	0.9936	0.053	0.8548	0.548	Anomalous
45 °C	0.9947	1.286	0.8589	0.010	0.8208	19.9	0.9320	0.037	0.8695	0.711	Anomalous

**Figure 3:**
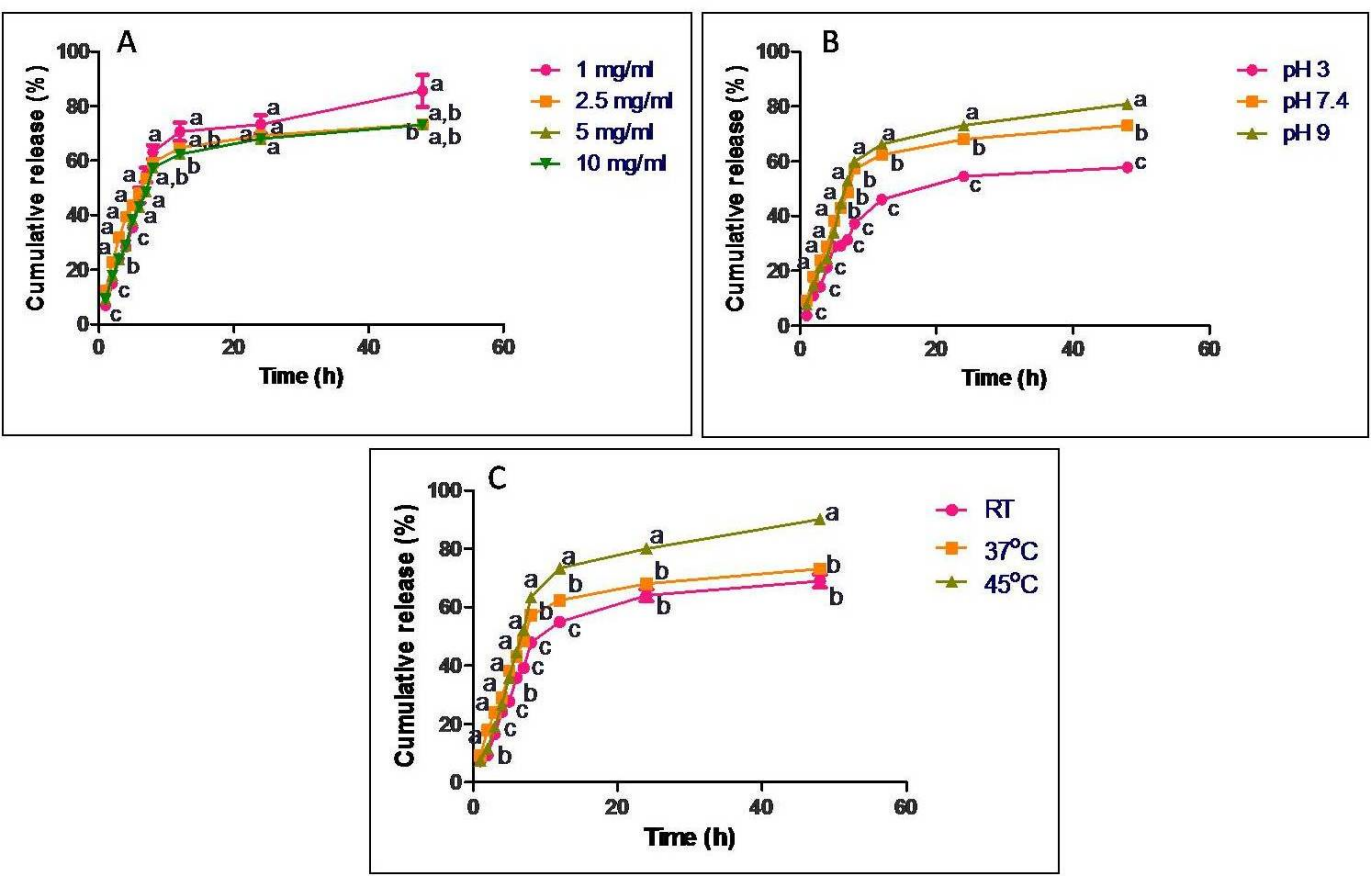
*In-vitro *release profile of GTP from casein nanoparticles at regular time intervals ranging from 1 h to 48 h under constant shaking conditions expressed as cumulative release percentage. Release studies were carried out with different concentrations of (A) GTP, (B) different pH and (C) different temperatures. Data are presented as means of three replicates. Means followed by same latter at each time interval are not significantly different at *p *≤ 0.05 according to DMRT

**Figure 4:**
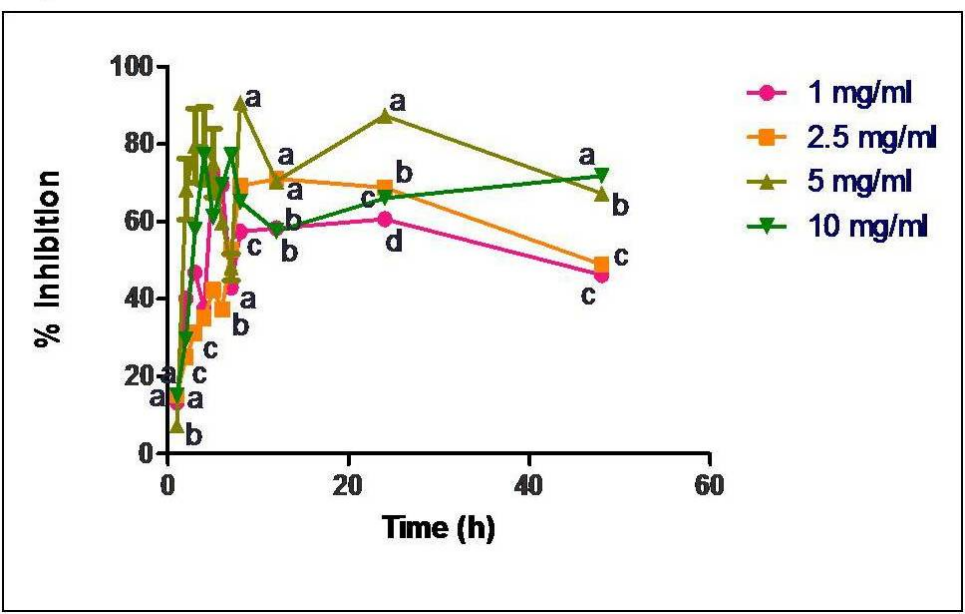
Radical scavenging activity of released samples of GTP loaded casein nanoparticles (loading concentrations of 1, 2.5, 5 and 10 mg/mL). Data are presented as means of three replicates. Means followed by same latter at each time interval are not significantly different at *p *≤ 0.05 according to DMRT

Encapsulation efficiency (EE) of GTP loaded casein nanoparticles was determined for GTP concentrations ranging from 1 to 10 mg/mL and is presented in Table 2. Highest EE (76.9%) was observed when GTP was used at a concentration of 5 mg/mL. Therefore, all other experiments were carried out using 5 mg/mL GTP.

*In-vitro* release of GTP from casein nanoparticles was studied on loading different concentrations of GTP (1, 2.5, 5 and 10 mg/mL) to casein nanoparticles, at different pH (pH 3, 7.4 and 9) and temperatures (25, 37 and 45 ºC) (Figure 3). When 1 mg/mL GTP was used for loading, about 63% of the drug was released in 8 h and a maximum of 85% was released in 48 h. On increasing the GTP concentration to 2.5 and 5 mg/mL, cumulative percentage of drug release was observed to be 75 and 72% respectively in 48 h. On further increasing the concentration of GTP to 10 mg/mL, about 82% release in 48 h was observed. Release was also influenced by pH of release medium. When pH was shifted from acidic to basic, there was an increase in the cumulative percentage of drug release. At pH3 about 57% of the drug was released in 48 h while at pH 7.4, about 72% and at pH 9 about 82% of the drug was released in 48 h. No significant difference was observed when release was carried out at 25 and 37 ºC, where about 68 and 73% was released in 48 h respectively but at 45 ºC about 90% of GTP was released in 48 h.

*In-vitro* release data obtained for all the parameters were fitted into various kinetic models to evaluate the mechanism of drug release. The regression parameters *i.*e., regression coefficients (R^2^) and release kinetic constants (k) are presented in Table 4 for different parameters. It was observed that all the release data showed good correlation with Zero order kinetics, except for that when GTP was used at a concentration of 2.5 mg/mL, which followed Hixson Crowell model of kinetics. Predominance in anomalous mode of release mechanism was observed for all the batches, except when GTP was used at a concentration of 2.5 mg/mL. The release exponent was observed to be 0.438, indicating a Fickian mode of drug release.

Radical scavenging activity of the released GTP samples were carried out through DPPH assay and the percentage inhibition was calculated. 

Free radical scavenging activity of the released GTP samples was observed to be about 88% in 4 h when GTP was used at a concentration of 5 mg/mL for encapsulation in casein nanoparticles (Figure 4).

## Discussion

Several studies have been published regarding the applications of casein based nano delivery systems. Livney reviewed the applications of milk proteins for their use as nanovehicles for the delivery of bioactives and the achievements of using casein nanovehicles for drug delivery systems in the recent past (22). All the studies showed promising results for using casein as a nanovehicle for the delivery of bioactive compounds. In our study, casein nanoparticles were prepared through desolvation technique using glutaraldehyde as a cross linker. GTP was used as a bioactive compound due to its several health benefits. The synthesised particles were characterized for their size and the surface charge (Table 2). Size of the particles plays a major role in delivering the drugs to the target site. In our study, larger size of the particles in DLS could be due to the aggregation of the particles in the dispersion medium, which was much higher for polyphenol loaded chitosan nanoparticles (average particle diameter being > 400 nm) and GTP loaded PLA-PEG nanoparticles (average particle diameter being > 300 nm) (24, 25). As observed in Table 2, the polydispersity index was shown to be the maximum at a GTP concentration of 10 mg/mL, which also might be due to aggregation of the particles as the concentration of GTP increased. ζ potential measurements were carried out for casein nanoparticles, before and after loading GTP and a difference in the surface charge was observed. As the concentration of GTP in the nanoparticles increased, a decrease in the ζ potential value was observed. This difference in the electric potential could be due to change in the protein conformation to protect the encapsulated GTP (23). All the GTP loaded nanopaticles showed ζ potential of -22.9 mV and above which would provide good stability, which showed a better result compared to the work carried out by Dube *et al*. (2010) on tea polyphenol loaded chitosan nanoparticles and for GTP loaded PLA-PEG nanoparticles (24, 25). 

To further confirm the entrapment of GTP in casein nanoparticles, molecular docking studies were carried out to identify the interactions between casein and the individual constituents of GTP. All the ligands showed good interactions with the target molecule and ECG showed highest binding energy (Figure 2, Table 3). 

Maximum EE was observed with 5 mg/mL GTP and on further increasing the concentration to 10 mg/mL, decrease in the EE was observed (Table 2). On comparing the EE of GTP to PLA-PEG nanoparticle, GTP loaded casein nanoparticles in our study showed good results with a maximum EE at a comparatively lower concentration of GTP (25). *In-vitro* release data for different concentrations of GTP showed no particular trend in the release of GTP and maximum amount of drug release was observed when 1 mg/mL GTP was encapsulated to casein nanoparticles (Figure 3). Thus, it could be said that the concentration of GTP entrapped and the cumulative percentage of drug release were independent. Drug release was further monitored at different pH conditions. When a drug is orally administered, it first passes through the gastro intestinal tract, where most of the drug is metabolized due to the harsh pH of the gastric juice. In our study, release of GTP was carried out in acidic conditions to mimic the conditions of the gastric juice. Although 57% of drug was released in acidic pH in 48 h, by the end of 4 h (equal to normal retention time of food in stomach) cumulative percentage of drug release was only 21%. Thus maximum amount of drug is retained within the nanoparticles and could be available for delivery to the site of action. Release studies were further carried out by altering the temperature. As the temperature increased, an increase in the cumulative percentage of drug release was observed which indicates the possible role of temperature in the release of GTP from casein nanoparticles. It has been reported that casein particles have no fixed structures and due to the lack of a rigid three dimensional structure, change in temperature may alter its structure (26). Thus, the observed increase in the cumulative percentage of drug release from casein nanoparticles at 45 ºC would be due to the alterations in the casein structure at a higher temperature.

Mathematical modelling of the released data showed that all release profiles have good correlation with Zero order reaction kinetics, where the concentration of drug release and time are independent (Table 4). Ideal model of drug release for nanoparticulate dosage forms or sustained release formulations is the Zero order kinetics (20). Few of the advantages of Zero order kinetics include a constant release rate as long as the drug is present in the core, no inhibitory build up of drug takes place in the dissolution medium, and a prolonged release is achieved, which is an ultimate goal for a controlled/ sustained release system (17). Therefore, as our data showed good correlation with Zero order reaction kinetics, casein nanoparticles could be used as an ideal carrier for the delivery of GTP, achieving a prolonged or a sustained release system. According to Korsmeyer-Peppas, a pure Fickian release has a release exponent n value limiting to 0.5, 0.45, and 0.43 for release from slabs, cylinders, and spheres (27). In our study n values ≤ 0.43 are considered as pure Ficikan release where the release is through diffusion and that ranging from 0.43-0.89 are considered as anomalous transport which involves a coupling of diffusion and erosion mechanism and that greater than 0.89 are referred to as super case II transport. The transport is characterized by polymer relaxation due to polymer erosion when enzymatic degradation occurs (28). When the relative relaxation time of the polymer is much shorter than the diffusion time, Fickian diffusion occurs. Once solvated, polymers assume an equilibrium state immediately, leading to polymer relaxation, known as case II transport. In many cases, transport mechanism leads to behaviour intermediate to Fickian and case II transport, referred to as anomalous transport (29). In our study, anomalous mode of drug transport was predominantly observed for all the batches and the mechanism of drug transport/release was observed to be a coupling of diffusion and erosion mechanism, where the rate of drug release is constant over time.

*In-vitro *antioxidant studies showed no particular trend in the activity for the released samples (Figure 4). Since GTP is known to be a good antioxidant source, there was a direct correlation between the concentration of GTP released and its antioxidant activity. As the concentration of drug release was maximum at about 4-5 h, proportionally there was an increase in the radical scavenging activity. Since all the released samples showed the scavenging activity, GTP loaded casein nanoparticles would serve as a potent antioxidant source.

## Conclusion

GTP encapsulated casein nanoparticles were prepared and characterized for the first time. *In-vitro *release showed that GTP loaded casein nanoparticles would serve as a good source for oral administration, as the release in acidic pH (simulated gastric juice/ stomach conditions) was much lower than the physiological and basic pH. All the release profiles showed good correlation with Zero order kinetics, which is known as an ideal model of release for nanoparticulate systems, showing anomalous mode of drug transport. Potent antioxidant activity was observed for all the released samples. All these observations indicate that casein nanoparticles could serve as a versatile vehicle for delivery of GTP.
